# Systematic Control of Self-Assembled Au Nanoparticles and Nanostructures Through the Variation of Deposition Amount, Annealing Duration, and Temperature on Si (111)

**DOI:** 10.1186/s11671-015-1084-z

**Published:** 2015-09-30

**Authors:** Ming-Yu Li, Mao Sui, Puran Pandey, Quanzhen Zhang, Eun-Soo Kim, Jihoon Lee

**Affiliations:** College of Electronics and Information, Kwangwoon University, Nowon-gu, Seoul 139-701 South Korea; Institute of Nanoscale Science and Engineering, University of Arkansas, Fayetteville, AR 72701 USA

**Keywords:** Self-assembled, Au nanoparticles, Deposition amount, Annealing duration, Annealing temperature, Ostwald-ripening, Volmer-Weber model, Si (111)

## Abstract

The size, density, and configurations of Au nanoparticles (NPs) can play important roles in controlling the electron mobility, light absorption, and localized surface plasmon resonance, and further in the Au NP-assisted nanostructure fabrications. In this study, we present a systematical investigation on the evolution of Au NPs and nanostructures on Si (111) by controlling the deposition amount (DA), annealing temperature (AT), and dwelling time (DT). Under an identical growth condition, the morphologies of Au NPs and nanostructures drastically evolve when the DA is only slightly varied, based on the Volmer-Weber and coalescence models: i.e. I: mini NPs, II: mid-sized round dome-shaped Au NPs, III: large Au NPs, and IV: coalesced nanostructures. With the AT control, three distinctive ranges are observed: i.e., NP nucleation, Au NPs maturation and melting. The gradual dimensional expansion of Au NPs is always compensated with the density reduction, which is explained with the thermodynamic theory. The DT effect is relatively minor on Au NPs, a sharp contrast to other metallic NPs, which is discussed based on the Ostwald-ripening.

**Electronic supplementary material:**

The online version of this article (doi:10.1186/s11671-015-1084-z) contains supplementary material, which is available to authorized users.

## Background

**Fig. 1:**
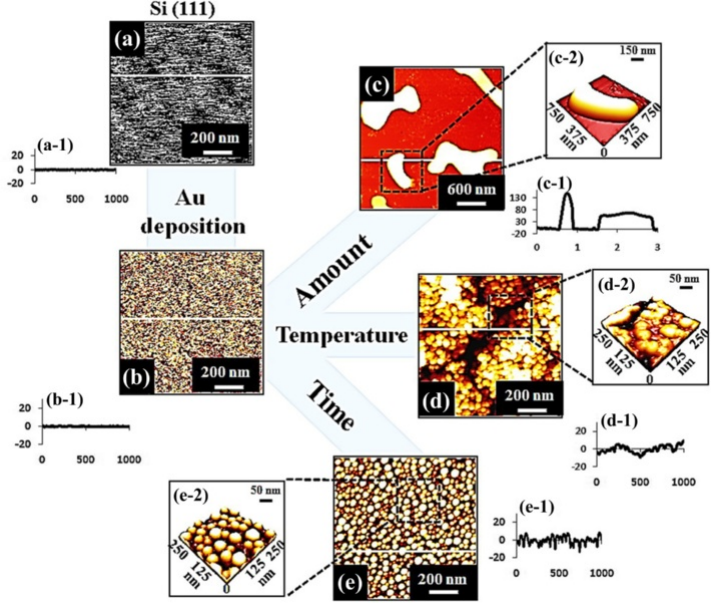
Illustration of the control of Au NPs and nanostructures on Si (111) by the annealing temperature (AT), deposition amount (DA), and dwelling time (DT) variation. **a** Bare Si (111) and **b** after Au deposition. (**c**–**e)** Au nanostructures fabricated with the DA, AT, and DT control. *a-1*–*e-1* Cross-sectional line profiles acquired from the white lines on AFM images of (**a–e**). *c-2–e-2* Corresponding AFM *side views* of the *boxed areas*

**Fig. 2:**
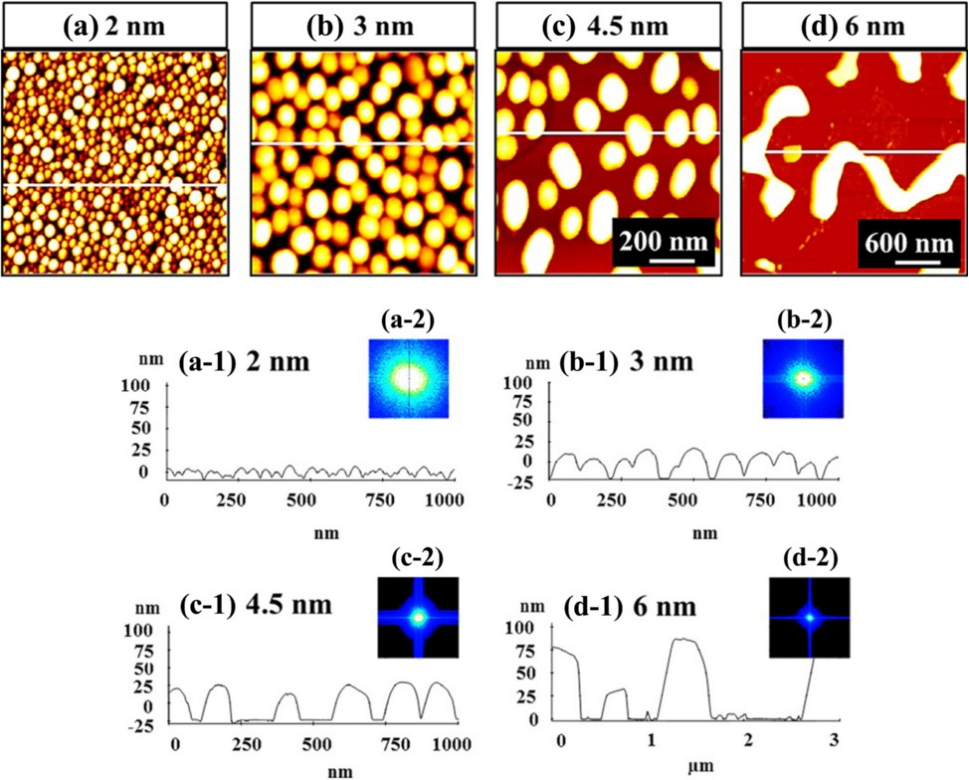
Control of the Au nanostructures from droplets to coalesced wiggly nanostructures by the systematic control of the DA on Si (111). **a**–**d** AFM *top views*. **a**–**c** 1 × 1 μm^2^ and **d** 3 × 3 μm^2^. Line profiles represent the surface cross-sectional information in (*a-1*–*d-1*). *a-2*–*d-2* 2D Fourier filter transform (FFT) power spectra

The size, density, and configurations of metallic nanoparticles (NPs) have played crucial roles in various applications, including the enhancement of device performances [1–10], nanowire (NW) fabrication [11–15], and nanoscale templates for various quantum nanostructures [16–25]. For example, Au NPs with a mesoscopic dimension (between 1 and 100 nm) have been widely adapted for the enhancement of the electron mobility [3, 4], light absorption [5, 6], and localized surface plasmon resonance [1, 2, 7–10], which thus can be witnessed with various sensors [1, 2, 7–9], field effect transistors [3, 4], and solar cells [5, 6]. In these devices, the red shift from the visible to the near-infrared region [1, 2], charge-storing capacity [3], conductance of electronic transport [4], enhancement of the reflective index sensitivity and scattering [7, 9, 10], and energy conversion efficiency [6, 8] can be efficiently explored and engineered with the variation of the size, density, and configurations of Au NPs. On the other hand, Au NPs can serve as a liquid phase nucleation medium for the NW synthesis based on the vapor-liquid-solid growth approach [11–15], and consequently, the orientation [11], shape [12], and density [13–15], of the NWs can be inherently determined by the Au NPs, offering a convenient approach to govern the NW utilization. Furthermore, based on the NP etching technique [16, 17], the metallic NPs can also be applied for the fabrication of the in situ nano-holed templates, based on which the size, configuration, and nucleation site controllable hybrid quantum- and nanostructures, such as quantum dots, molecules [18–21], and rings [22–24], can be fabricated subsequently via the NP epitaxy. Especially, with the size control of Au NPs, conical nano-pore etching has been successfully achieved on Si, potentially a convenient and simple approach to the voltage-gated switching device fabrication [25]. Consequently, the systematic investigation on the shape, configuration, and density control of Au nanostructures can offer a valuable basis for the relevant applications, however, which still relatively lacks by now. On the other hand, the fabrication of self-assembled nanoparticles has been achieved via chemical [26] and physical [27] methods as a convenient and effective way to control the nanostructure synthesis. Thus, in this paper, approaches to the fabrication of Au NPs and nanostructures on Si (111) via the systematic control of the deposition amount (DA), annealing temperature (AT), and dwelling time (DT) are studied. Figure 1 illustrates an overview of the effect of the DA, AT, and DT on the self-assembled Au nanostructure synthesis. Depending on the DA, the Au structure undergoes drastic shape evolution in four phases: I) mini NPs, II) mature NPs, III) lateral growth, IV) coalescence, which can be explained by the coalescence model along with the Volmer-Weber (V-W) growth model [28–31]. With the variation of the DA, the wiggly Au nanostructures can be successfully fabricated instead of the round dome-shaped NPs, as shown in Fig. 1c, (c-2). Depending on the AT, the evolution from the nucleation of tiny particles to the well-defined round dome-shaped Au NPs was clearly demonstrated. Finally, the surface melting was observed at the higher temperatures than 800 °C due to the eutectic point of the Si-Au alloy as shown in Fig. 1d, (d-2). The size and density of the self-assembled Au NPs can be conveniently engineered via the AT variation, which can be related to the enhanced diffusion based on the thermodynamics. Throughout the DT range, the rounded dome-shaped Au NPs exhibited relatively small changes in size and density, which was described by the Ostwald-ripening [32–34].

## Methods

In this work, samples were specifically prepared to investigate the effects of annealing temperature (AT), deposition amount (DA), and dwelling time (DT) on Au NPs on Si (111). For substrates, 4-inch p-type singular-Si (111) with a thickness of 1000 μm were diced into 1 × 1 cm^2^ in a dicing saw and treated with the RCA clean. Prior to the deposition of gold, samples were degassed at 850 °C for 30 min under 1 × 10^−4^ Torr in a pulsed laser deposition (PLD) system to get rid of surface oxide and other contaminants. Additional file 1: Figure S1 shows the Raman spectra of the bare Si surface excited with a CW diode-pumped solid-state (DPPS) laser of a wavelength of 532 ± 1 nm with an output power of 120 mW. For the variation of DA, in general, Au was deposited at a rate of 0.05 nm/s with the ionization current of 3 mA in a plasma ion-coating chamber under 1 × 10^−1^ Torr and the ionization time was varied to achieve each DA (0.25, 0.5, 1, 2, 3, 4, 4.5, 5, 5.5, 6, 8, and 16 nm). After the Au deposition, surface morphologies of samples were relatively smooth, as clearly shown in Additional file 2: Figure S2. For the investigation of the DT effect on Au NPs, samples were systematically annealed at a pre-determined temperature of 700 °C with a fixed DA of 2 nm for 30 s up to 5 h, respectively. Samples were annealed by a halogen lamp at a ramping rate of 2.3 °C/s under a chamber pressure of 1 × 10^−4^ Torr. The procedure was operated by a computer controlled recipe. In order to investigate the evolution of Au NPs depending on the AT, similarly 2 nm of Au was equally deposited on the substrate and the samples were annealed with the variation of AT from 50 to 850 °C for a fixed annealing duration of 30 s. With the termination of each growth recipe, the temperature was quenched immediately to get rid of the Ostwald-ripening [32–34]. Subsequent to the sample synthesis, for the smaller area characterization, the surface morphology of each sample was performed by an atomic force microscope (AFM) with a non-contact mode, and for larger area characterization, a scanning electron microscopy (SEM) was used. The Si AFM tip had a length of ~125 μm with a radius of less than 10 nm. The spring constant and the resonant frequency of the tip used were ~40 N/m and ~170 kHz (NSC16/AIBS, μmasch), respectively. The AFM cantilever was back-side coated with ~30 nm Al to enhance the reflection of laser and thus improving the sensitivity of scanning by a factor of ~2.5. Additionally, the characterization of surface morphology was performed by the same type of tips from a single batch to minimize the tip effect and for the consistency of the analysis. For the analysis of the obtained AFM data, 2-D Fourier Filter Transform (FFT) power spectra, line profiles, and surface area ration (SAR) were methodically analyzed by using XEI software (Park Systems). The Fourier filter transform (FFT) power spectra was obtained by converting the height information from the spatial domain to the frequency domain after Fourier filter transform and shows the height distribution along with the directionality. In addition, the SAR [*γ*] is roughness in % considering the surface area (*x* × *y*) [*α*] in 2D and geometric area (*x* × *y* × *z*) [*β*] in 3D, given by $\gamma =\left(\frac{\beta\ \hbox{--} \alpha\ }{\beta}\right)\times 100\ \left[\%\right]$. The elemental characterization was performed by an energy-dispersive X-ray spectroscopy (EDS) system with the spectral and mapping modes (Thermo Fisher Noran System 7) under vacuum.

## Results and Discussion

**Fig. 3:**
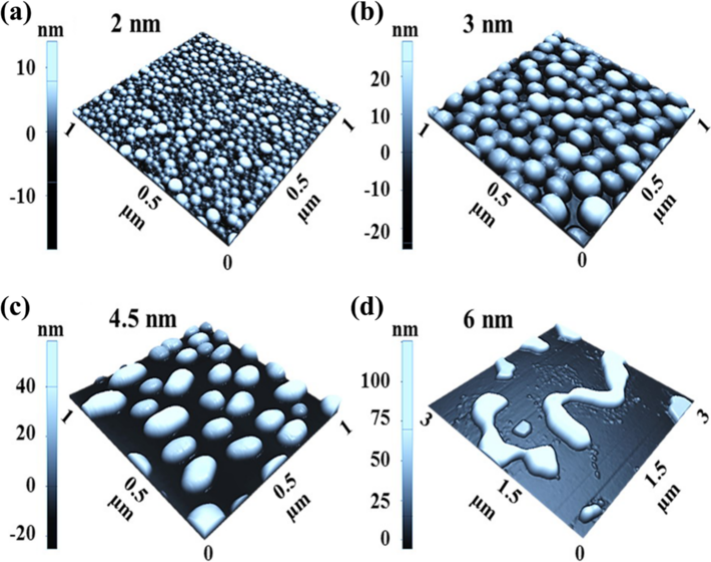
Three-dimensional (3D) AFM *side views* of the self-assembled Au nanostructures fabricated with the DA variation on Si (111). Au nanostructures were fabricated under the fixed annealing temperature (AT) at 700 °C and the dwelling time (DT) for 30 s. **a**–**d** AFM *side views*. **a**–**c** 1 × 1 μm^2^ and **d** 3 × 3 μm^2^. The *scale bars* indicate height

**Fig. 4:**
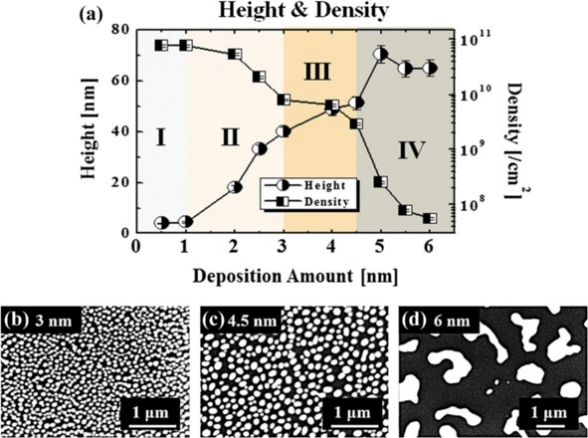
**a** Summary plot of the average height (AH) and average density (AD) of Au nanostructures as a function of DA on Si (111). *Error bars*: ±5 %. **b**–**d** Scanning electron microscope (SEM) images of the larger scale areas of the Au nanostructures with 3, 4.5, and 6 nm DA. SEM images 4 (*x*) × 2.78 (*y*) μm^2^ in (**b**) and (**c**) and 6.7 (*x*) × 4.64 (*y*) μm^2^ in (**d**)

Figures 2, 3, and 4 summarize the deposition amount (DA) effect on Au nanostructures on Si (111) between 0.5 and 6 nm at a fixed annealing temperature (AT) of 700 °C for a fixed dwelling time (DT) of 30 s. Generally, depending on the DA, a drastic evolution in the configuration and size of Au nanostructures in four distinctive phases was witnessed, which suggests that the Au nanostructures very sensitively respond to the slight modification of the DA, as shown in Figs. 2, 3, and 4. The evolution of Au nanostructures can be described with the coalescence growth model along with the V-W model [28–31]. According to the V-W, the *E*_I_ < *E*_Au_: namely, the binding energy between Au adatoms (*E*_Au_) is stronger than the one between Si atoms and Au adatoms (*E*_I_). With a sufficient thermal energy supplied, the Au adatoms can spontaneously diffuse to form nuclei at the relatively lower energy sites and once the nuclei are formed, the adatoms can be absorbed to form 3D islands (NPs) with the stronger binding energy (*E*_I_ < *E*_Au_). Being provided with additional Au atoms within a diffusion length with the increased DA, the Au NPs tend to absorb nearby Au adatoms to increase dimension in order to maintain the equilibrium in this thermodynamic system. Gradually, the lateral increase in the dimension of Au NPs becomes much more noticeable than the vertical growth until reaching the critical radius (<*R*_D_>), which can be expressed by ${R_{\mathrm{D}}}^4\approx \frac{D_{\mathrm{S}}\gamma {\Omega}^{4/3}}{kT}{D}_{\mathrm{C}}$ [29, 30], where *D*_C_ is the critical DA, *γ* is the surface free energy, the Ω is the atomic volume of Au, *k* is the Boltzmann constant, *T* is the absolute temperature, and *D*_S_ is the diffusion coefficient as a function of *T*. Given that the AT has been fixed under the increased DA, as soon as the radius of Au NPs reached the <*R*_D_>, the coalescence can begin to occur and the wiggly nanostructures can form instead of the round dome-shaped NPs. With further increased DAs, even a layer can be expected to form along with the preferential lateral coalescence. As described, the evolution of Au nanostructures based on the coalescence and V-W growth models in the dimension and configuration is clearly presented in Figs. 2 and 3. Initially, the round dome-shaped Au NPs with well-packed density were fabricated with 2 nm DA in Figs. 2a and 3a. With only additional 1 nm more DA, the dimension of the Au NPs was expanded both laterally and vertically as shown in Figs. 2b and 3b. At 4.5 nm, the vertical size expansion of Au NPs became slower likely due to reaching the <*R*_D_>, and the lateral growth became more obvious, which resulted in the presence of elongated Au NPs, as in Figs. 2c and 3c. Then, the Au NPs gradually coalesced into wiggly nanostructures along with the increased DAs, as evidenced by the coalescence occurring at 6 nm in Figs. 2d and 3d. As sharply compared in the cross-sectional line profiles in Fig. 2(a-1), (d-1), the height of resulting nanostructures were radically increased with the increased DAs from 2 to 6 nm. The morphological evolution can also be observed with the Fourier filter transform (FFT) power spectra in Fig. 2(a-2), (d-2). Initially, at 2 nm the spectrum covered almost 90 %, but, with the increased DAs, the spectra gradually shrunk into a small spot due to the reduced frequencies. To specify the DA effect on the size and density of Au NPs, the average height (AH) and average density (AD) of the Au nanostructures with each DA are well summarized in Fig. 4a. Depending on the configurations of Au nanostructures, four phases (I–IV) are introduced in the plot. Phase I: mini NPs below 1 nm; the mini NPs were densely packed over the surface and the density was ~8 × 10^10^ cm^−2^. Phase II: mid-sized round dome-shaped Au NPs between 1 and 3 nm. The NPs showed a sharp height expansion of 9.1 times, which was compensated by the density decrease of 89.6 %. Phase III: large Au NPs between 3 and 4.5 nm deposition. The increment of the AH of Au NPs became slower by only 1.3 times, and the elongated NPs began to show up, indicating the <*R*> was around the <*R*_D_> as shown in Figs. 2b and 3b. The elongation becomes much more pronounced with further increased DAs, and finally at around 6 nm DA, the coalesced wiggly nanostructures appeared, the phase IV. In addition, the nanostructures appeared quite uniform regardless of the DAs as shown by the scanning electron microscope (SEM) images in Fig. 4b, d.

**Fig. 5:**
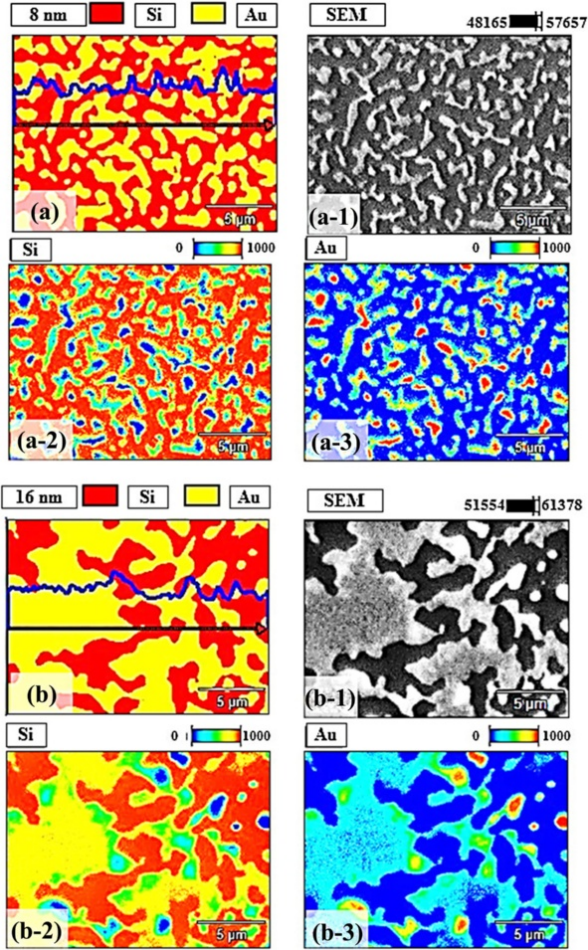
Energy-dispersive X-ray spectroscopy (EDS) phase maps of the Au nanostructures with the 8 nm DA in (**a)** and 16 nm DA in (**b). a**–**b** Combined phase maps of Si and Au. The line profiles show the Au components along the *arrow. a-1–b-1* Corresponding SEM images: 20 (*x*) × 15 (*y*) μm^2^. Si phase maps are shown in (*a-2*) and (*b-2*) and Au phase maps are shown in (*a-3*) and (*b-3*), respectively

**Fig. 6:**
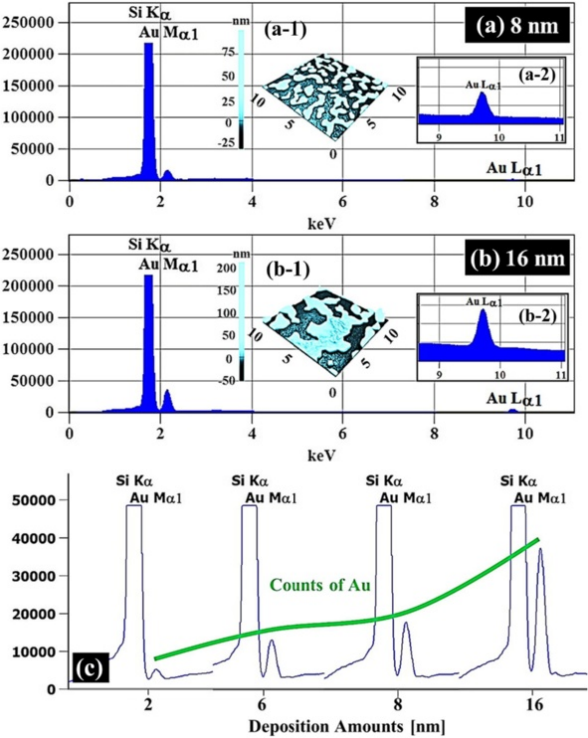
Energy-dispersive X-ray spectroscopy (EDS) spectra of the samples with 8 nm DA in (**a)** and 12 nm in (**b)** on Si (111). Insets in (*a-1*) and (*b-1*) show the corresponding AFM *top views. a-2–b-2* Enlarged spectral range between 9–11 KeV. **c** Evolution of the Au Mα1 peaks as a function of DA

Figure 5 shows the energy-dispersive X-ray spectroscopy (EDS) phase maps of the Au nanostructures with the 8 nm DA in Fig. 5a and 16 nm DA in Fig. 5b and the EDS spectra along with the DA variation are presented in Fig. 6. The 3D EDS phase maps of the Au nanostructures with the 8 nm and 16 nm DA are shown in Additional file 3: Figures S3 and Additional file 4: S4. With further increased DAs, the coalesced nanostructures grew much longer and wider and finally formed layered structures, as shown with the SEM images in Fig. 5(a-1), (b-1). In the combined phase maps of Si and Au in Fig. 5a, b, the red and yellow colors clearly show the elements distribution of Au and Si, and the evolution of the coalesced Au nanostructures based on the coalescence growth model are correspondingly revealed. Separated phase maps of Si and Au are shown in Fig. 5(a-2), (a-3) and (b-2), (b-3), respectively. The change in the Au contents can be evidently observed in the EDS spectra as shown in Fig. 6. The presence of Au Mα1 peak at 2.123 keV with 16 nm deposition was around twice of the 8 nm sample, likely due to the enhanced interaction between Au and X-ray. As a result, the Lα1 peak at 9.711 keV of Au was also appeared to be much more pronounced with the 16 nm as shown in Fig. 6(a-2), (b-2). As can be expected, regardless of DAs, the Kα peaks of Si at 1.740 keV appeared uniform. Figure 6c summarizes the evolution of the Au Mα1 peaks as a function of DA, and the increase in Au counts is clearly shown. Overall, the Au NPs and nanostructures were quite sensitive to the DA modification and the drastic evolution of Au nanostructures from the mini NPs to the coalesced nanostructures was clearly observed throughout the DA range. The coalescence growth based results can be also witnessed with the Au NPs synthesized on the polystyrene, polymethyl methacrylate [28], TiO2 [29], and glass [31] and with the Pt NPs on SiO2 [30]. Meanwhile, interestingly for the Ag [35], Au [36], and Pd/Pt [37] NPs fabricated on alumina, GaAs, and Si showed a gradual dimensional increase up to 100 nm without the formation of layer, which can be regarded as the V-W dominant growth.

**Fig. 7:**
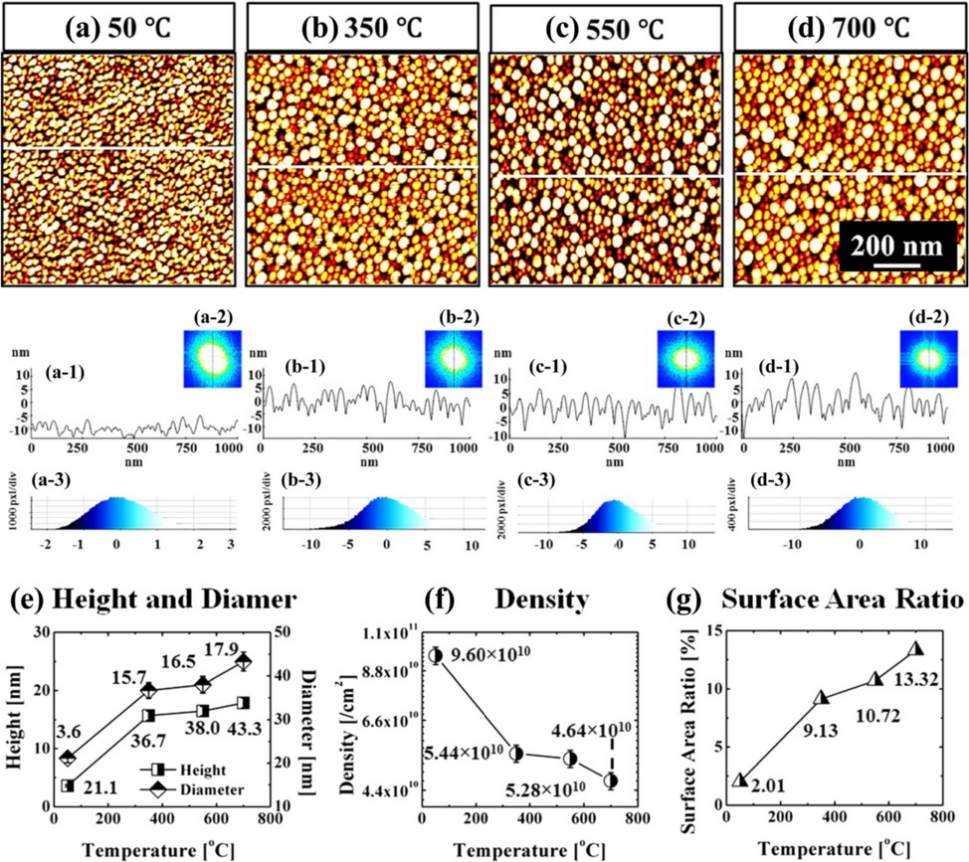
Fabrication of the self-assembled Au droplets by the control of the AT on Si (111). The AFM *top views* in (**a**–**d**) are 1 × 1 μm^2^. The cross-sectional line profiles in (*a-1*–*d-1*), FFT power spectra in (*a-2–d-2*), and the height distribution histograms (HDH) in (*a-3–d-3*) are presented, respectively. The plots show the summary of the AH and LD in (**e**), the AD in (**f**), and the surface area ratio (SAR) in (**g**). The surface area [*α*] is (*x* × *y*), the geometric area [*β*] is (*x* × *y* × *z*), and the SAR is {(*β* − *α*)/*β*] × 100 [%]}. *Error bars*: ±5 %

**Fig. 8:**
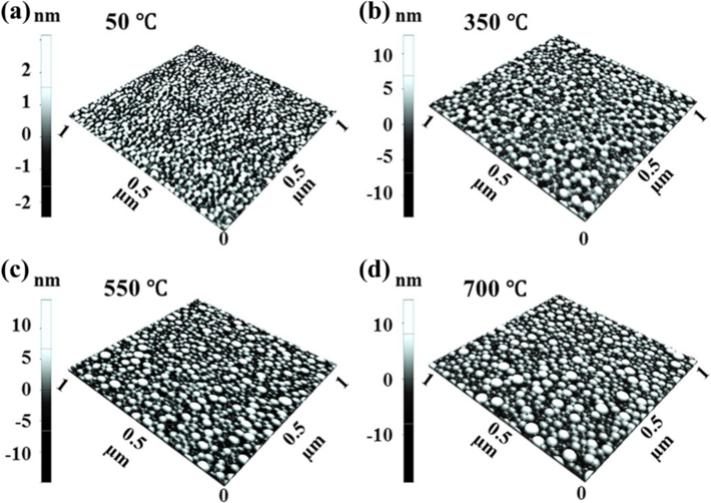
3D AFM *side views* of the self-assembled Au droplets fabricated with the AT variation between 50 and 700 °C on Si (111). Au droplets were fabricated by the systematic control of the AT under a fixed DA of 2 nm and the DT for 30 s. **a**–**d** AFM *side views* of 1 × 1 μm^2^

**Fig. 9:**
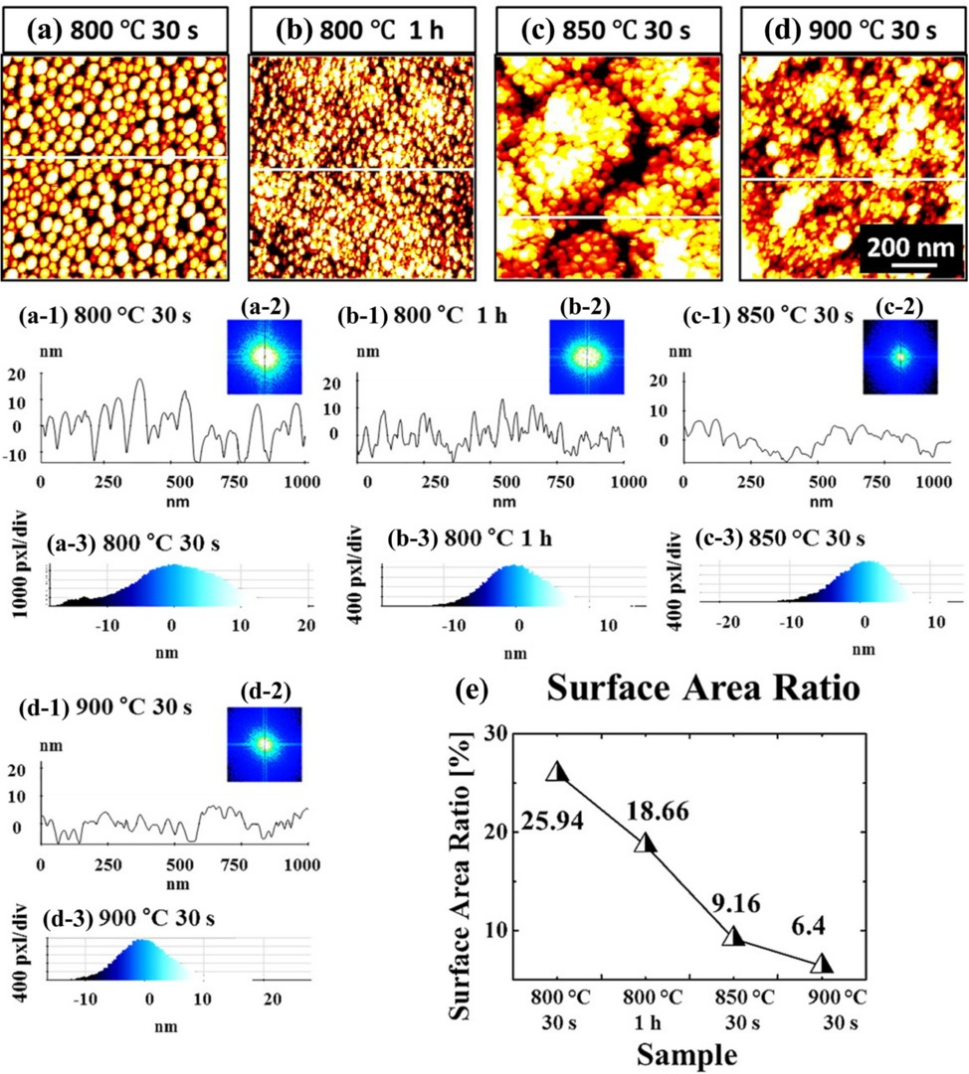
Mophologic evolution of the self-assembled Au droplets on Si (111) at relatively higher temperatures between 800 and 900 °C. The DT and AT are indicated with the labels. **a**–**d** AFM *top views* of 1 × 1 μm^2^. *a-1*–*d-1* Cross-sectional line profiles. *a-2*–*d-2* FFT power spectra. *a-3–d-3* HDH. **e** SAR

**Fig. 10:**
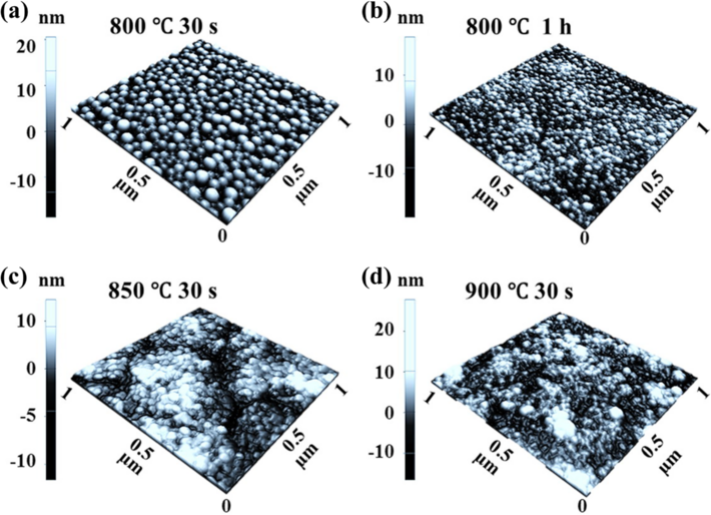
3D AFM *side views* of the surface morphologies at relatively higher ATs between 800 and 900 °C. Each sample was deposited with a fixed DA of 2 nm. **a**–**d** AFM *side views* of 1 × 1 μm^2^

Figures 7 and 8 show the evolution of self-assembled Au NPs by varying the annealing temperature (AT) between 50 and 700 °C with a fixed DA of 2 nm on Si (111). In general, gradually increased size of the self-assembled Au NPs was observed from the nucleation of small NPs to the well-defined larger NPs and for the compensation, the AD was gradually decreased as a function of AT. This evolution trend can be explained based on the thermal diffusion theory. The diffusion length (*l*_D_) can be given as ${l}_{\mathrm{D}}=\sqrt{D_{\mathrm{S}}t}$, where *t* is the diffusion time of adatoms and the diffusion coefficient (*D*_S_) is a direct constant of the surface temperature (*T*_S_). With the increased AT, the Au adatoms can obtain increased thermal energy to diffuse further, in other words, the increased *l*_D_. Given that *E*_I_ < *E*_Au_ at increased ATs, the nucleus can absorb more adatoms with longer *l*_D_ and this can result in the increased dimension of Au NPs. For example, at the TA as low as 50 °C, the tiny Au NPs were fabricated after the formation of nuclei as in Figs. 7a and 8a. The Au NPs were with quite small dimension: ~3.6 nm in the AH and ~21.13 nm in the LD and with correspondingly high density of ~9.6 × 10^10^ cm^−2^. This can be considered as the nucleation of Au NPs, which is still quite interesting that the Au adatoms respond to that low thermal energy at ~50 °C. To remind, the melting point of pure Au is 1064.18 °C and that of Si is 1414 °C. The reason of the adatom diffusion at very low thermal energy can be likely due to the fact that the eutectic point of Si/Au is indeed much lower at ~370 °C for 2.85 wt% of Au [35–37] and the very thin nanoscale Au layer was utilized. At the AT of 350 °C, the round dome-shaped Au NPs were utilized as in Figs. 7b and 8b. The AH and LD were increased by ×4.36 to ~15.68, and ×1.74 to ~36.69 nm. Meanwhile, the AD was decreased by ×1.76 to 5.44 × 10^10^ cm^−2^ in order to compensate the size expansion. Between 350 and 700 °C, the change of the dimension and density of NPs was somewhat minor as clearly seen in the plots and the AH and LD reached 17.9 and 43.3 nm respectively and the corresponding AD was 4.46 × 10^10^ cm^−2^ at 700 °C with a decent NP uniformity, as indicated by the FFT spectrum. This can suggests that decent round dome-shaped Au NPs can be fabricated between 350 and 700 °C on Si (111). Figure 7(a-3), (d-3) shows the height distribution histograms (HDHs), indicating the size increment of Au NPs along with the increased AT. At 50 °C, the HDH ranged between ~±2 nm, as in Fig. 7(a-3), and along with the AT increased, the HDHs were gradually expanded and finally ranged between ~±10 nm at 700 °C, as in Fig. 7(b-3), (d-3). As in Fig. 7g, the SAR was also gradually increased from ~2 to 13 %, and this should be rooted from the dimensional increase of the NPs. In brief, the nucleation of Au NPs occurred at relatively very lower temperature of 50 °C and decent round dome-shaped Au NPs can be fabricated between 350 and 700 °C. During the evolution of Au NPs, the increased size was always compensated by the decreased density as a function of AT, which can be regarded as a rather general behavior of metallic NPs on various semiconductors substrates [38–46], such as on the GaN [38], sapphire [39, 40], and GaAs [41–44]. Similar to the Au NPs, the Ag [38, 39], Ga [40–42], and In NPs [43, 44] also tended to sacrifice their density for the dimensional increase regardless of the variation of the DA or AT. At higher ATs above 800 °C, the self-assembled Au NPs can only be synthesized by a very short time annealing, such as 30 s and the extended dwelling time (DT) or AT can trigger surface melting as shown in Figs. 9 and 10. For instance, as shown in Figs. 9a and 10a, round domed Au NPs can successfully be fabricated at 800 °C for the annealing of 30 s. However, the surface started to melt and mix with the Au and formed some bulks at longer DTs as shown in Figs. 9b and 10b. On the other hand, higher temperature directly resulted in much damaged surface morphologies as evidenced between 850 and 900 °C. The change of surface morphology is also clearly depicted in cross-sectional line profiles in Fig. 9(a-1), (d-1). Correspondingly, the melting of Au NPs also caused shapely decrease in the SAR from 25.94 to 6.4 nm by over four times as in Fig. 9e. This surface melting can be caused by much lower eutectic point of Si/Au as discussed [47–49]. Likely, the extended DT can accelerate the intermixing of the Au and Si atoms to form Si-Au alloy and similarly higher temperature can also drive the formation of alloy at much higher rate.

**Fig. 11:**
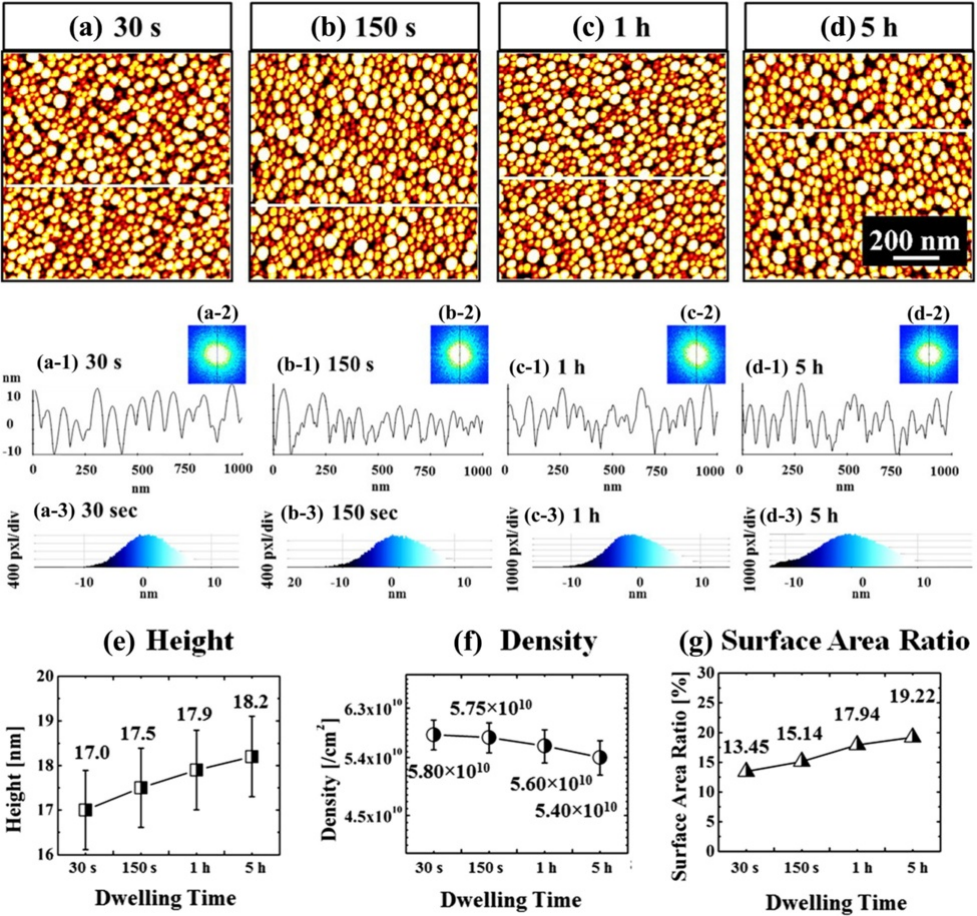
Evolution of the self-assembled Au droplets by the control of the DT on Si (111). **a**–**d** AFM images *top views* 1 × 1 μm^2^. *a-1–d-1* Cross-sectional line profiles. *a-2–d-2* FFT power spectra. *a-3–d-3* HDH. Plots summarize the AH in (**e)**, the AD in (**f)**, and SAR in (**g)** as a function of DT. *Error bars*: ±5 %

Figure 11 and Additional file 5: Figure S5 summarize the dwelling time (DT) effect on the self-assembled Au droplets on Si (111). The Au NPs were fabricated with a variation of DT between 30 s and 5 h under an identical growth condition (2 nm at 700 °C) and the corresponding AH, AD, and SAR are summarized in Fig. 11e, g. Overall, the self-assembled Au NPs gradually evolved with the increased DT as witnessed with the dimensional evolution; however, the effect of extended DT on the self-assembled NPs appeared to be relatively minor. The AHs of Au NPs were between ~17 and 18 nm and the ADs were ~5 × 10^10^ cm^−2^ and the SAR showed a few % change in Fig. 11e, g. Meanwhile, the HDHs of the Au NPs ranged between ~±10 nm for all samples as shown Fig. 11(a-3), (d-3). Nevertheless, with extended annealing duration, the gradual increase in the SAR and AH was equally observed. The dimensional increase as a function of dwelling time can be based on the Ostwald-ripening, and epitaxilly fabricated NPs showed similar behaviors [32, 45, 46]. According to Ostwald-ripening theory, the <*R*> is driven by dwelling time *t*: $<R(t){>}^4-<{R}_0{>}^4=\frac{8{N}_0{D}_S\gamma {\Omega}^2}{45{k}_B Tl(L)}t$, [47–49] where <*R*_0_> is the radius of NPs at *t* = 0. The density of nucleation sites (*N*_0_) is 1.22 × 10^15^/cm^2^, the interface energy between Au and air (*γ*) is 1.5 J/m^2^, the Au atomic volume (Ω) is 1.69 × 10^−29^ m^3^, and the characteristic length (*L*) is about 3. Being provided with the favorable diffusion at sufficient thermal energy with the stronger binding energy (*E*_I_ < *E*_Au_), larger Au NPs can keep absorbing nearby adatoms and this results in the increase in the <*R*> at extended *t*. The <*R*> would keep increasing with the extended *t* until reaching the critical <*R*_C_> of NPs. As mentioned, the dimensional change was relatively mild in this experiment, perhaps, it could be due to the fact that the thermal energy is relatively high at 700 °C and the DA was small with 2 nm and thus the resulting NPs are small. Therefore, the Au NPs might have already nearly reached <*R*_C_> during the increase of the target substrate temperature.

## Conclusions

In this study, the systematic approaches to engineer Au NPs and nanostructures on Si (111) via the control of the deposition amount (DA), annealing temperature (AT), and dwelling time (DT) are successfully demonstrated and discussed. For the control of the DA with the fixed AD and DT (700 °C for 30 s), four distinctive phases depending on the configurations of Au nanostructures were clearly observed based on the coalescence growth model along with the V-W model: I: mini NPs, II: mid-sized round dome-shaped Au NPs, III: large Au NPs, and IV: coalesced nanostructures. In addition, the gradual dimensional expansion was always compensated by the density reduction, which were systematically discussed with the summary plots, cross-sectional line profiles, HDHs. EDS maps and SEM images proved the formation of Au layer based on the coalescence growth model. For the effect of AT, the ATs from near the room temperature up to 900 °C were tested and the results showed three ranges: Au NP nucleation below 350 °C, decent NPs formation with the decent uniformity between 350 and 750 °C, and surface melting above 800 °C, which have been discussed and explained with the thermodynamic theory. For the effect of the DT with the fixed the DA and AT (2 nm at 700 °C), 30 s to 5 h were tested. With the DT variation, the change was discussed based on the Ostwald-ripening.

## Additional files

Additional file 1: Figure S1. Raman spectra of bare Si (111) at room temperature. The emission was excited by a CW diode-pumped solid-state (DPPS) laser of a wavelength of 532 ± 1 nm with an output power of 120 mW. The signal was detected by a TE cooled CCD detector. **a** Full range between 154 and 1388 cm^−1^. Each Si peak is indicated with blue arrows. **b** Enlarged area between 260 and 700 cm^−1^ to show transverse acoustical (TA) mode peaks at 303 and 619 cm^−1^. The transverse optic (TO) peak at Γ appeared at 520 cm^−1^. The full width at half maximum of TO is 11.6 cm^−1^. Two TO peaks were also detected at 939 and 985 cm^−1^ [50]. Additionally, the peak at 619 cm^−1^ is also associated with Si [51]. (JPG 102 kb) Additional file 2: Figure S2. Variation of the deposition amount (DA) between 0.25 and 6 nm on Si (111). Each DA is labeled on the top of the corresponding AFM top views in (**a**–**j**). Line profiles in (a-1–j-1) are acquired from the white lines in (**a**–**j**) showing smooth cross-sectional surface morphologies at each DA. (a-2–j-2) 2D FFT power spectra. (JPG 476 kb) Additional file 3: Figure S3. Three-dimensional (3D) EDS phase maps of the wiggly Au nanostructures with the 8 nm DA on Si (111). *Color scale bars* indicate the counts between 0 and 1000. **a** Side view of the 3D phase map for Au. **b** Top view of the 3D phase map for Au. **c**–**d** Side and top views of the 3D phase maps for Si. (JPG 301 kb) Additional file 4: Figure S4. 3D EDS phase maps of the Au nanostructures with the 16 nm DA on Si (111). *Color scale bars* indicate the counts between 0 and 1000. **a** Side view of the 3D phase map of Au. **b** Top view of the 3D phase map of Au. **c**–**d** Side and top views of the 3D phase maps for Si. (JPG 278 kb) Additional file 5: Figure S5. 3D AFM side views of the self-assembled Au droplets on Si (111) at various DTs: **a** 30 s, **b** 150 s, **c** 1 h, and **d** 5 h. Au droplets were annealed at 700 °C. **a**–**d** AFM 3D side views of 1 × 1 μm^2^. (JPG 228 kb)
