# Supplementary figures and images for: Systematic Control of Self-Assembled Au Nanoparticles and Nanostructures Through the Variation of Deposition Amount, Annealing Duration, and Temperature on Si (111)
Source: Nanoscale Res Lett. 2015 Sep 30;10:380. doi: 10.1186/s11671-015-1084-z (PMC4883281; doi:10.1186/s11671-015-1084-z)

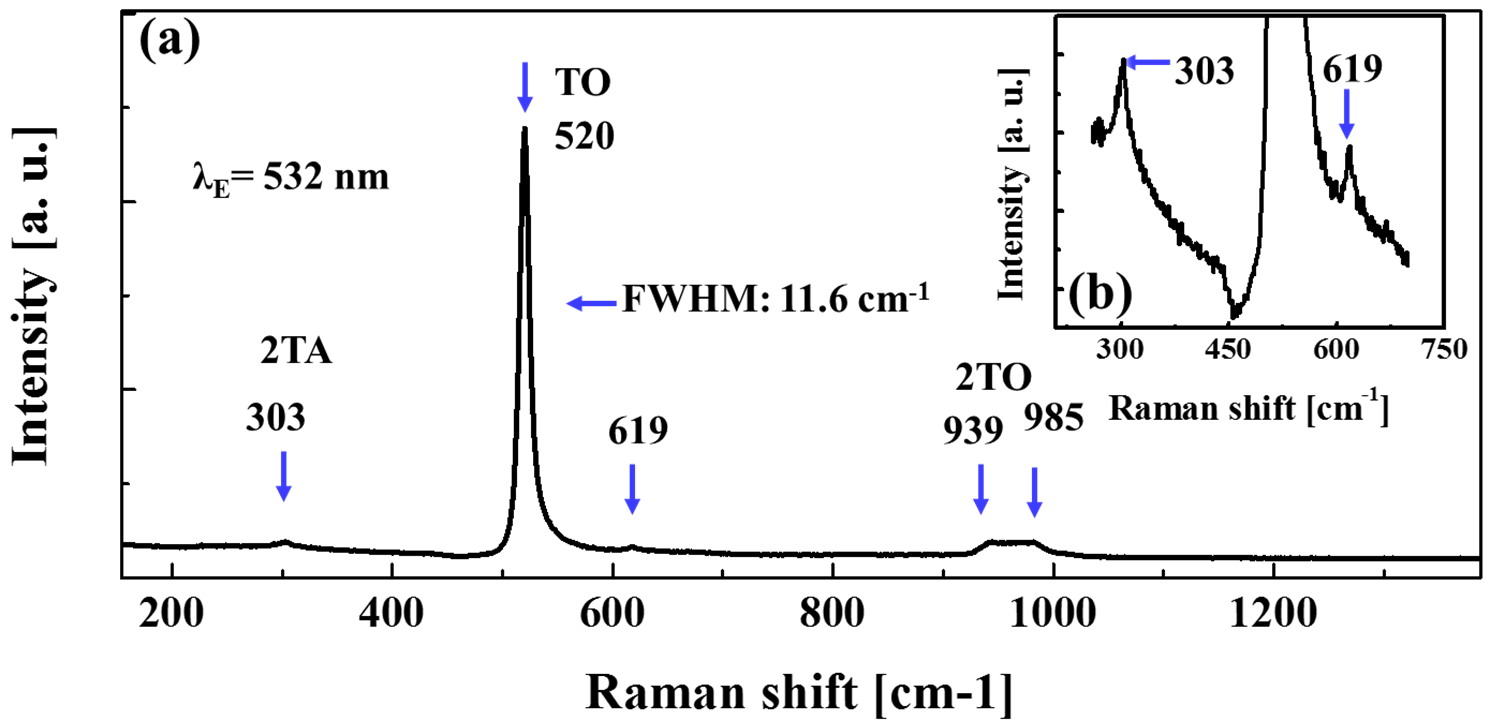

Supplement: Additional file 1: Figure S1. — Raman spectra of bare Si (111) at room temperature. The emission was excited by a CW diode-pumped solid-state (DPPS) laser of a wavelength of 532 ± 1 nm with an output power of 120 mW. The signal was detected by a TE cooled CCD detector. a Full range between 154 and 1388 cm−1. Each Si peak is indicated with blue arrows. b Enlarged area between 260 and 700 cm−1 to show transverse acoustical (TA) mode peaks at 303 and 619 cm−1. The transverse optic (TO) peak at Γ appeared at 520 cm−1. The full width at half maximum of TO is 11.6 cm−1. Two TO peaks were also detected at 939 and 985 cm−1 [50]. Additionally, the peak at 619 cm−1 is also associated with Si [51]. (JPG 102 kb) [file 11671_2015_1084_MOESM1_ESM.jpg]

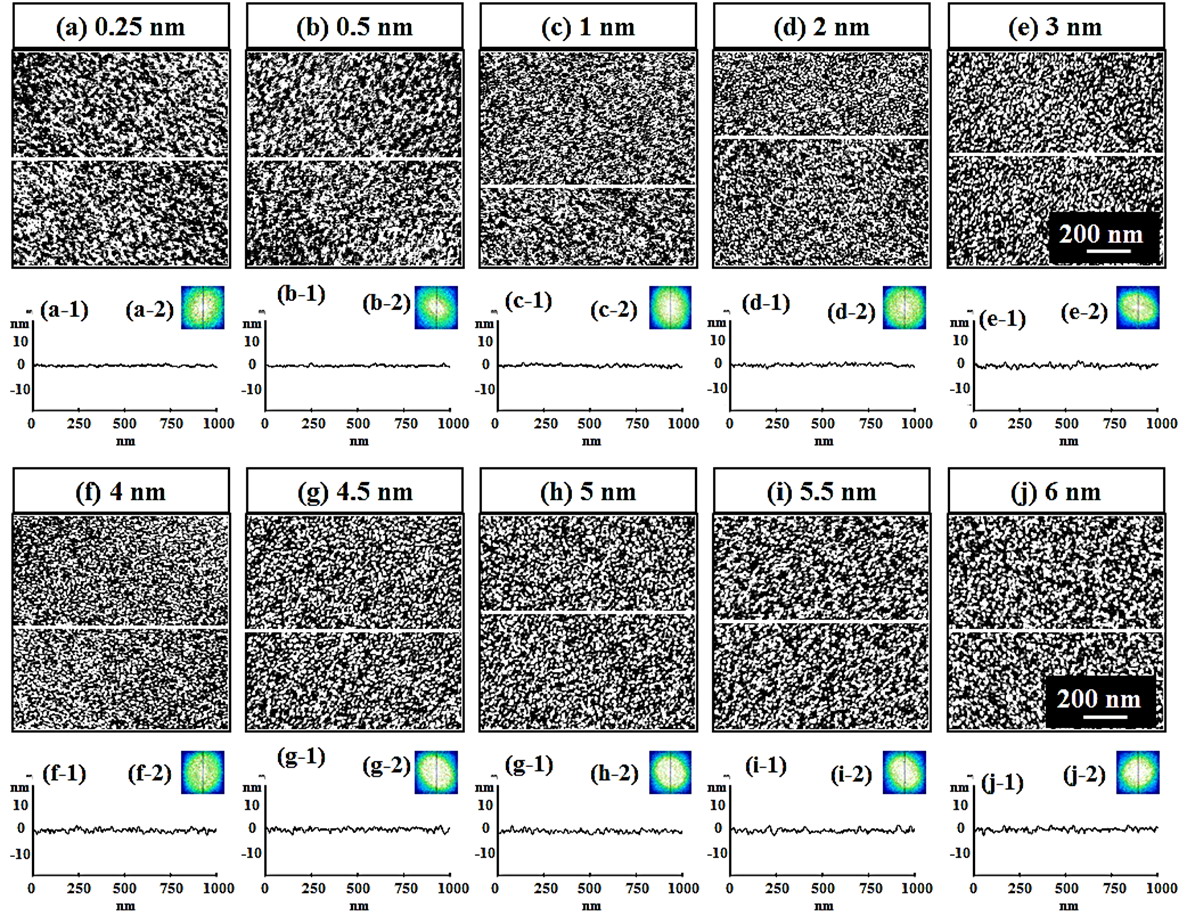

Supplement: Additional file 2: Figure S2. — Variation of the deposition amount (DA) between 0.25 and 6 nm on Si (111). Each DA is labeled on the top of the corresponding AFM top views in (a–j). Line profiles in (a-1–j-1) are acquired from the white lines in (a–j) showing smooth cross-sectional surface morphologies at each DA. (a-2–j-2) 2D FFT power spectra. (JPG 476 kb) [file 11671_2015_1084_MOESM2_ESM.jpg]

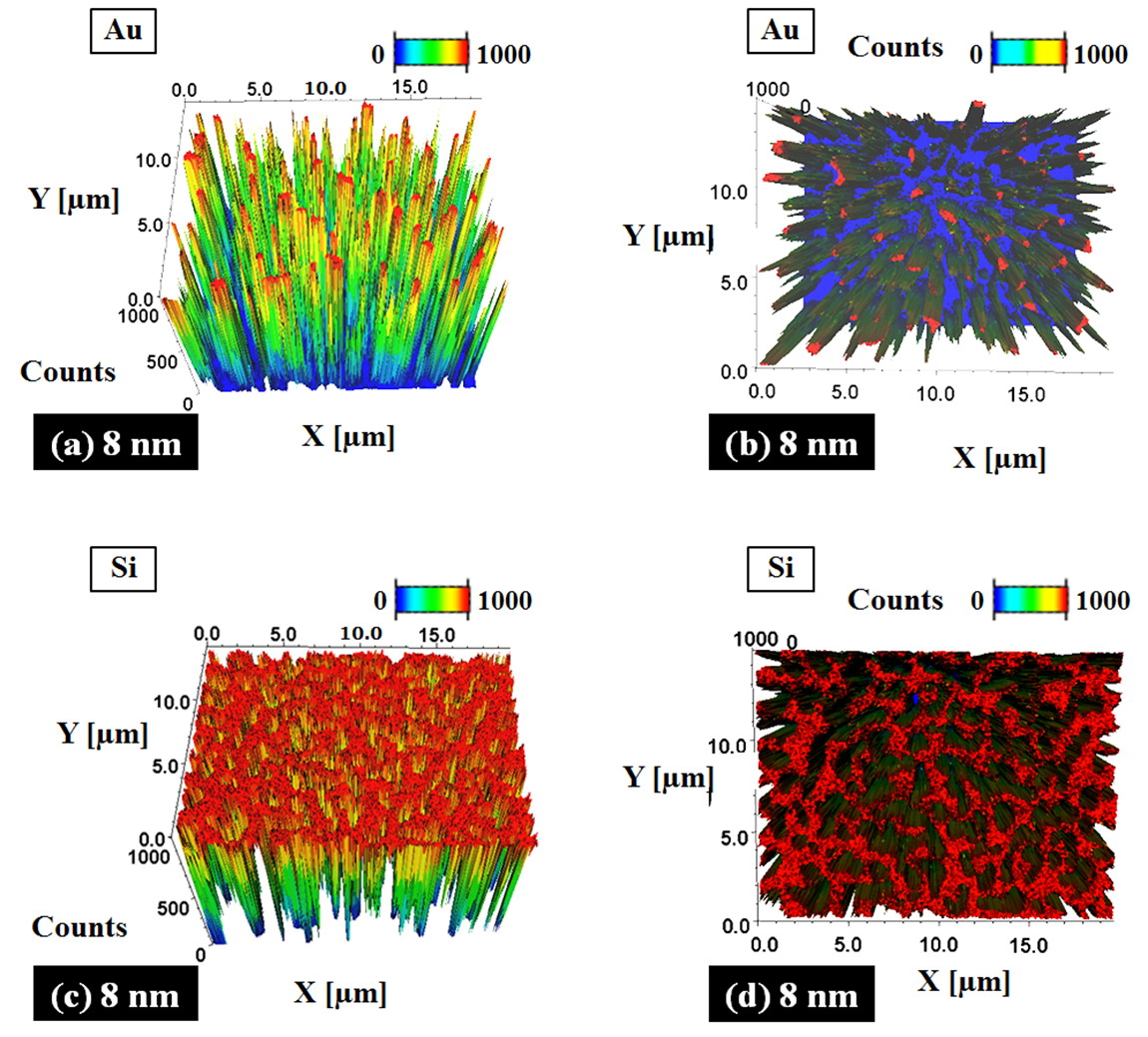

Supplement: Additional file 3: Figure S3. — Three-dimensional (3D) EDS phase maps of the wiggly Au nanostructures with the 8 nm DA on Si (111). Color scale bars indicate the counts between 0 and 1000. a Side view of the 3D phase map for Au. b Top view of the 3D phase map for Au. c–d Side and top views of the 3D phase maps for Si. (JPG 301 kb) [file 11671_2015_1084_MOESM3_ESM.jpg]

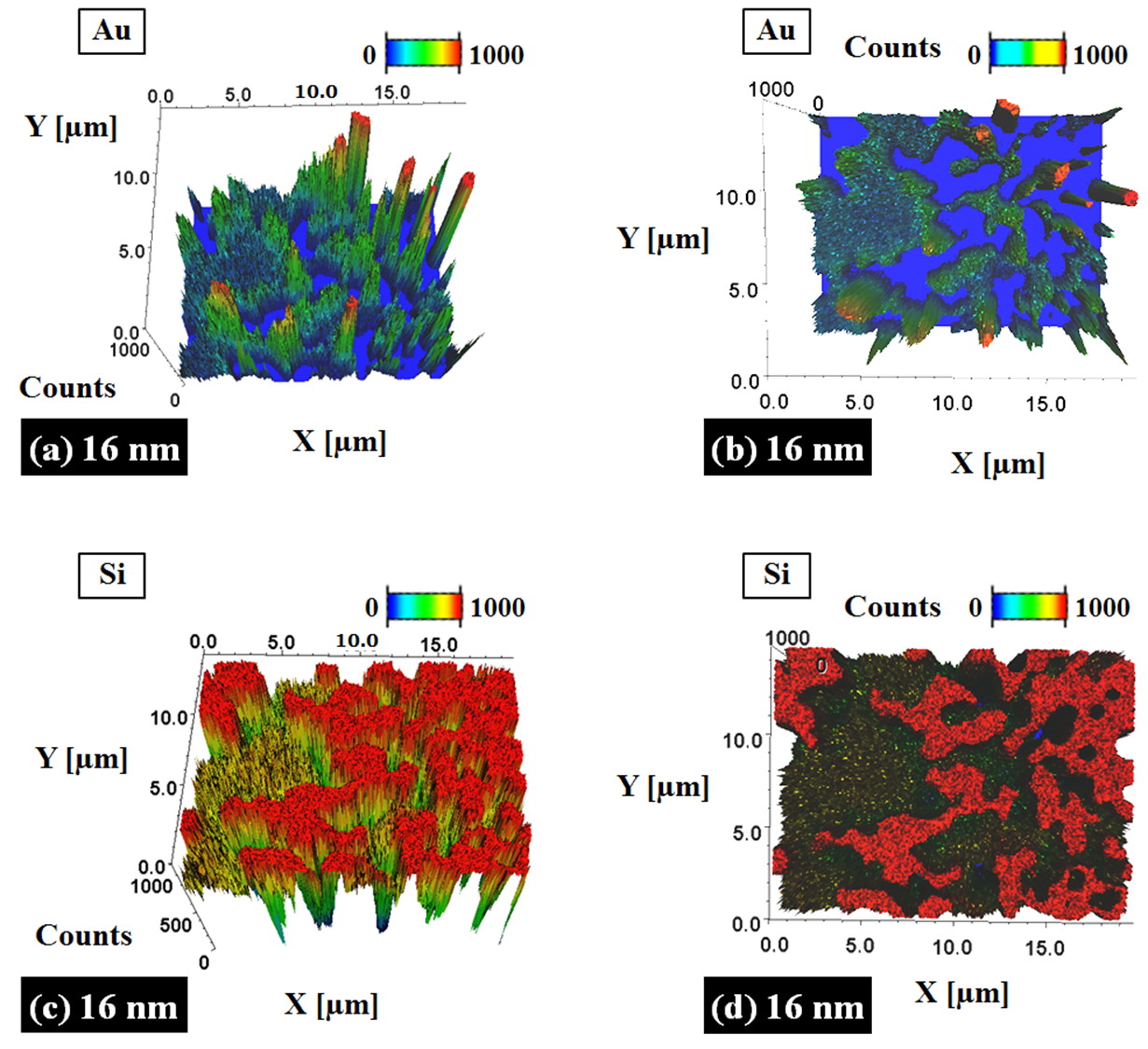

Supplement: Additional file 4: Figure S4. — 3D EDS phase maps of the Au nanostructures with the 16 nm DA on Si (111). Color scale bars indicate the counts between 0 and 1000. a Side view of the 3D phase map of Au. b Top view of the 3D phase map of Au. c–d Side and top views of the 3D phase maps for Si. (JPG 278 kb) [file 11671_2015_1084_MOESM4_ESM.jpg]

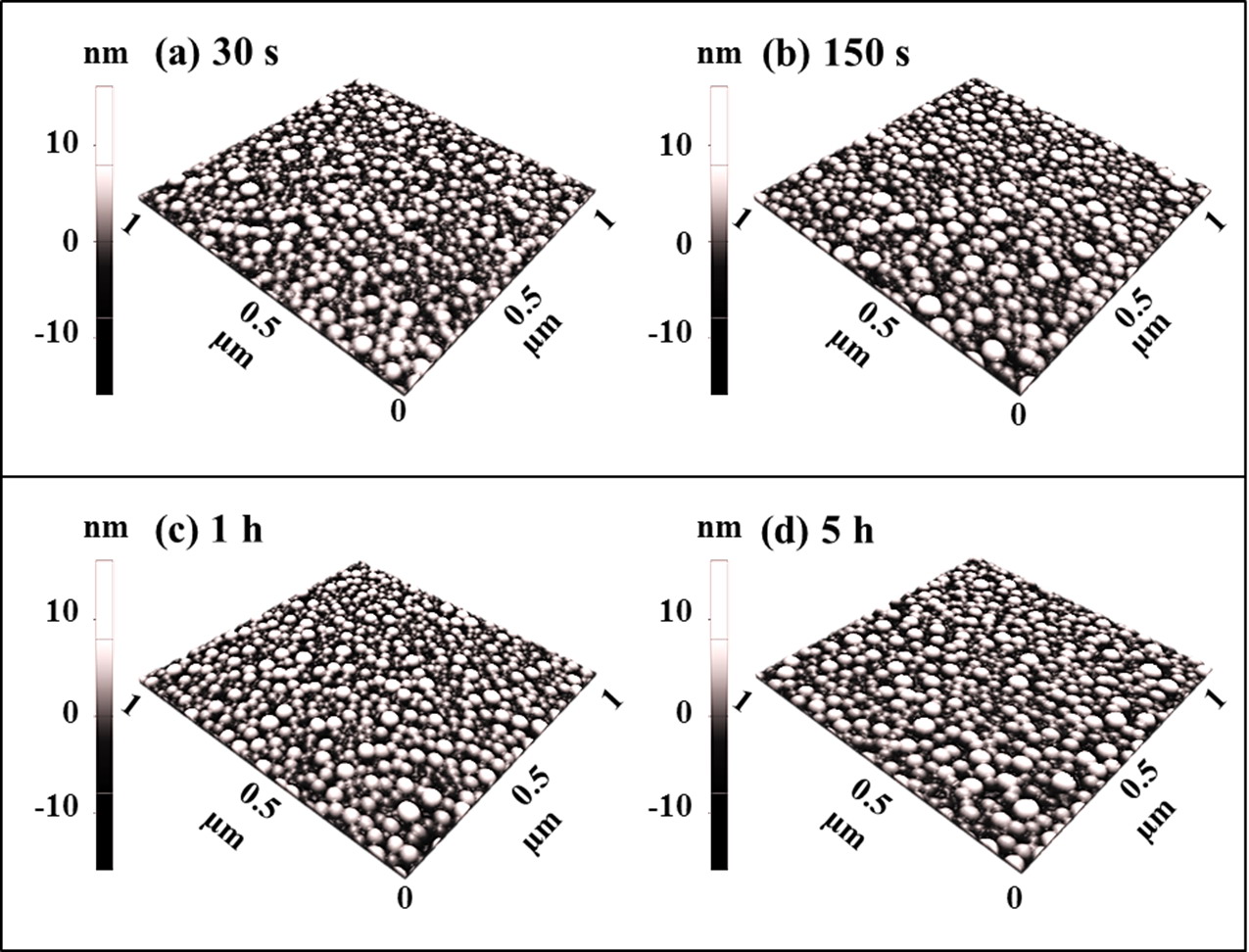

Supplement: Additional file 5: Figure S5. — 3D AFM side views of the self-assembled Au droplets on Si (111) at various DTs: a 30 s, b 150 s, c 1 h, and d 5 h. Au droplets were annealed at 700 °C. a–d AFM 3D side views of 1 × 1 μm2. (JPG 228 kb) [file 11671_2015_1084_MOESM5_ESM.jpg]
